# Computed tomography-guided percutaneous biopsy of head and neck masses: techniques, outcomes, and complications

**DOI:** 10.1590/0100-3984.2020.0100

**Published:** 2021

**Authors:** Mauricio Kauark Amoedo, Chiang Jeng Tyng, Paula Nicole Vieira Pinto Barbosa, Rayssa Araruna Bezerra de Melo, Maria Fernanda Arruda Almeida, Rubens Chojniak, Almir Galvão Vieira Bitencourt

**Affiliations:** 1 Department of Imaging, A.C.Camargo Cancer Center, São Paulo, SP, Brazil.

**Keywords:** Image-guided biopsy, Needle biopsy, Tomography, X-ray computed, Head and neck neoplasms, Biópsia guiada por imagem, Biópsia por agulha, Tomografia computadorizada, Neoplasias de cabeça e pescoço

## Abstract

**Objective:**

To assess the technique, efficacy, and safety of computed tomography (CT)-guided percutaneous biopsies of head and neck masses.

**Materials and Methods:**

This was a retrospective, single-center study of CT-guided percutaneous core-needle biopsies of head and neck masses. For the analysis of diagnostic accuracy, biopsy results were compared with the final diagnosis, which was determined by histological examination and clinical follow-up.

**Results:**

We evaluated 74 biopsies performed in 68 patients. The mean age of the patients was 55.6 years. Most of the lesions (79.7%) were located in the suprahyoid region, and the maximum diameter ranged from 11 mm to 128 mm. The most common approaches were paramaxillary (in 32.4%), retromandibular (in 21.6%), and periorbital (in 14.9%). Five patients (6.8%) developed minor complications. The presence of a complication did not show a statistically significant association with any clinical, radiological, or procedure-related factor. Sufficient material for histological analysis was obtained in all procedures. Thirty-eight biopsies (51.4%) yielded a histological diagnosis of malignancy. There was a false-negative result in three cases (8.3%), and there were no false-positive results. The procedure had a sensitivity of 92.7%, a specificity of 100%, and an accuracy of 96.0%.

**Conclusion:**

Our results demonstrate that CT-guided percutaneous core-needle biopsy of head and neck lesions is a safe, effective procedure for obtaining biological material for histological analysis.

## Abstract (pt)

**Objetivo:**

Avaliar a técnica, eficácia e segurança das biópsias percutâneas guiadas por tomografia computadorizada (TC) de lesões de cabeça e pescoço.

**Materiais e Métodos:**

Este estudo retrospectivo e unicêntrico incluiu pacientes submetidos a biópsia percutânea guiada por TC de lesões de cabeça e pescoço. Para avaliação da acurácia diagnóstica, os resultados da biópsia foram comparados com o diagnóstico final determinado por avaliação histológica ou acompanhamento clínico.

**Resultados:**

Foram avaliadas 74 biópsias realizadas em 68 pacientes. A média de idade dos pacientes foi de 55,6 anos. A maioria das lesões (79,7%) estava localizada na região supra-hioide e o maior diâmetro variou de 11 a 128 mm. Os acessos mais comuns utilizados foram paramaxilar (32,4%), retromandibular (21,6%) e periorbital (14,9%). Cinco pacientes (6,8%) desenvolveram complicações menores e não houve associação estatisticamente significante entre a presença de complicações e fatores clínicos, radiológicos ou relacionados ao procedimento. Foi obtido material suficiente para análise histológica em todos os casos. Trinta e oito biópsias (51,4%) tiveram diagnóstico histológico de malignidade. Houve três (8,3%) resultados falso-negativos e nenhum falso-positivo, demonstrando sensibilidade de 92,7%, especificidade de 100% e acurácia de 96,0%.

**Conclusão:**

Nossos resultados demonstram que a biópsia percutânea guiada por TC de lesões de cabeça e pescoço é um procedimento seguro e efetivo para obter material para análise histológica.

## INTRODUCTION

Percutaneous biopsies of head and neck masses have been considered safe and effective when the target is in a superficial location. Ultrasound is the imaging method of choice for the guidance of biopsies of superficial head and neck lesions, such as those located in the thyroid gland or salivary glands. Its advantages are availability, real-time monitoring of needle positioning, absence of exposure to ionizing radiation, and low cost. However, because of the limited acoustic window for visualization of a target interposed between bone and air-filled structures^(1)^, ultrasound is less useful for the evaluation of deep masses. In that setting, computed tomography (CT) provides excellent spatial resolution and has therefore been increasingly used in clinical practice^(2)^.

Fine-needle aspiration (FNA) is often the procedure of choice for the diagnosis of head and neck masses. Some studies have assessed CT guidance techniques for deep masses, reporting diagnostic rates of approximately 90% and accuracy of 86-88%^(3,4)^. However, core-needle biopsies have been shown to yield larger tissue samples than does FNA, allowing immunohistochemical studies and more accurate histological analysis^(4,5)^. One of the few studies comparing the two techniques showed that, for the diagnosis of malignant lesions, the accuracy of core-needle biopsy was 100%, significantly higher than the 93% found for FNA^(6)^. Although little data are available in the literature, core-needle biopsy of head and neck masses has become established as a safe, effective technique for obtaining representative tissue samples^(1)^. However, CT-guided head and neck biopsies account for only 1% of all CT-guided interventions and many of these procedures are canceled or aborted due to the lack of a safe window^(7)^.

The objective of this study was to assess the technique, efficacy, and safety of CT-guided percutaneous core-needle biopsies of head and neck masses performed at a cancer center.

## MATERIALS AND METHODS

This was a retrospective, single-center study of patients who underwent CT-guided percutaneous core-needle biopsy of head and neck masses at a cancer center between March 2012 and December 2016. The study was approved by the institutional research ethics committee, and all participating patients gave written informed consent.

The following variables were collected through a review of patient medical records, as well as from CT images and procedure reports: gender; age; history of cancer (primary diagnosis, if known, and previous treatments); imaging characteristics of the target masses (dimensions and location); technique (needle gauge, approach, and need for intravenous contrast); complications (early and late); results of histopathological examination of the biopsy specimens (specimen quality having been evaluated by pathologists and considered adequate if the quantity of material collected was sufficient for histological analysis); need for a new biopsy; clinical outcome after biopsy (surgery, chemotherapy, radiotherapy, watchful waiting, or a combination of those).

### Procedures

Prior to the procedure, a targeted history was taken to obtain pertinent clinical information, such as the indication for biopsy, history of cancer, previous treatments, and current medications (discontinuation of anticoagulant agents was mandatory), and the results laboratory test (coagulation panel and complete blood count) were reviewed, an international normalized ratio < 1.5, platelet count > 50,000, and hemoglobin levels > 8.0 g/dL were mandatory). All biopsies were performed by an experienced interventional radiologist or by a radiologist in training under supervision of the attending radiologist. Biopsy planning was based on recent images clearly showing the target lesion and on images acquired at the time of the procedure, with contrast administration if necessary.

Each procedure was performed in one of two CT scanners: a single-slice helical scanner (GE HiSpeed; GE Medical Systems, Milwaukee, WI, USA); or a 16-slice scanner (Brilliance CT 16; Philips Healthcare, Best, The Netherlands). Biopsies were performed by using the coaxial technique, with 15- to 19-gauge needles, followed by placement of a 16- to 20-gauge core needle (except in bone biopsies, for which specific needles were used). All procedures were performed under local anesthesia and intravenous sedation. In most cases, if there were no immediate complications, there was no need for follow-up imaging after the procedure. In the absence of symptoms, patients were discharged after 1 h of observation. Follow-up imaging was reserved for patients who had experienced complications (such as local bleeding) during the procedure and for those who developed potentially procedure-related symptoms during the observation period. The approaches employed were divided, according to the recommendations of previous studies^(8,9)^, into two categories (suprahyoid and infrahyoid), as detailed below.

#### Suprahyoid

**Figure 1.**
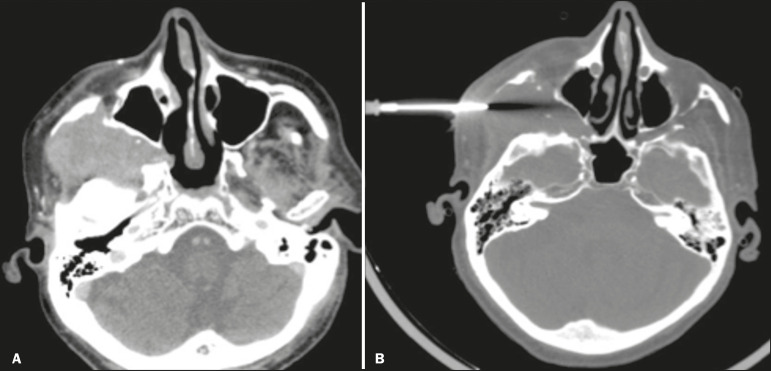
Biopsy of an infratemporal mass. The patient was a 63-year-old man with a history of oropharyngeal squamous cell carcinoma. The lesion was located in the right infratemporal space (**A:** axial CT view). Biopsy was performed through an infrazygomatic approach (**B**). The histopathological findings were consistent with meningioma.

*Infrazygomatic (infratemporal)*-For target lesions located at the base of the skull, including the pterygopalatine fossa and the masticator space (Figure 1), as well as the parapharyngeal, pharyngeal, retropharyngeal, and prevertebral spaces.

*Retromandibular*-For target lesions in the deep parotid, parapharyngeal, pharyngeal mucosal, and lower retropharyngeal spaces, as well as the carotid space (if it is medially deviated by the target lesion).

**Figure 2.**
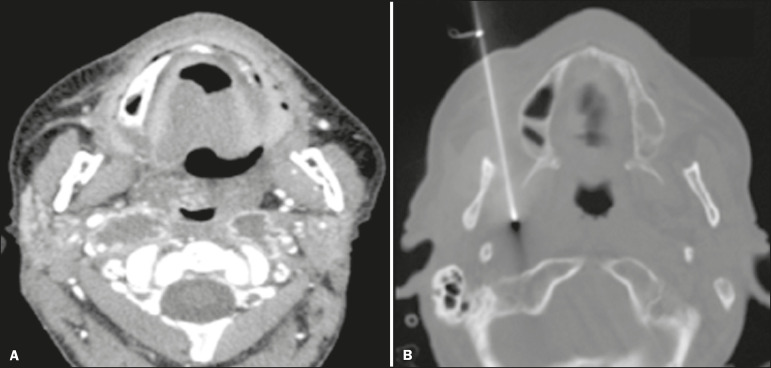
Biopsy of a parapharyngeal mass. The patient was a 54-year-old man with a history of moderately differentiated oropharyngeal squamous cell carcinoma treated with surgery and radiotherapy. Follow-up imaging revealed suspicious bilateral cervical lymph node enlargement in the parapharyngeal spaces (**A**). Superficial lymph node aspiration biopsies were negative for malignancy. Due to progression of lymphadenopathy during follow-up, the patient underwent core-needle biopsy through a right paramaxillary approach (**B**), which revealed secondary neoplastic involvement.

*Paramaxillary (retromaxillary or buccal)*-For target lesions in the infrazygomatic portions of the masticator space and posterior portions of the parapharyngeal spaces (Figure 2), as well as the pharyngeal mucosal, carotid, and deep parotid spaces, also being useful to provide access to lateral portions of the retropharyngeal space, the prevertebral portion of the paravertebral space, the anterior arch of the atlas (C1 vertebra), the odontoid process, the body of the axis (C2 vertebra), and the foramen ovale (with cranial angulation of the needle).

*Submastoid*-For target lesions in the carotid space (when the vessels are medially displaced), anterolateral portions of the paravertebral space (when the carotid space is shifted anteriorly), and, occasionally, the parapharyngeal space.

*Transoral*-For target lesions in the posterior pharyngeal space, retropharyngeal space, and prevertebral portion of the paravertebral space, as well as in the anterior portions of the C1 and C2 vertebrae, including the odontoid process.

**Figure 3.**
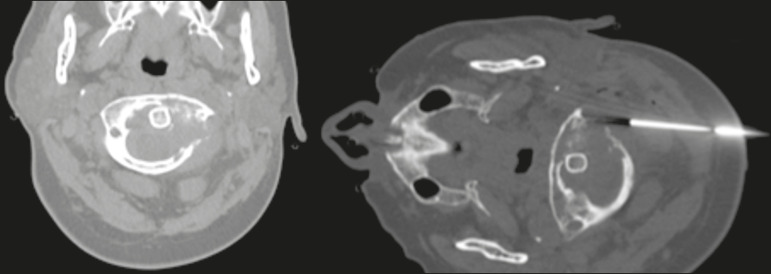
Biopsy of a vertebral-body mass affecting the C1 vertebra. The patient was a 48-year-old woman with breast cancer and a known lytic lesion of the left lamina of the C1 vertebra. Percutaneous biopsy through a posterior approach revealed metastatic cancer.

*Posterior*-For target lesions involving the spinous processes, laminae, or articular processes of the upper cervical vertebrae or the occipital condyle, also potentially being useful for masses involving the lateral portions of the C1 and C2 vertebrae (Figure 3).

**Figure 4.**
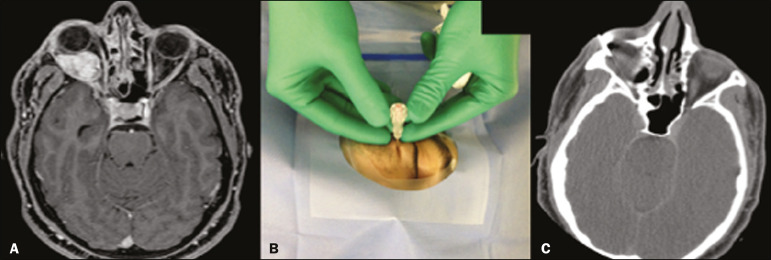
Biopsy of a retro-orbital mass. The patient was a 30-year-old man with mild proptosis secondary to a rapidly growing space-occupying lesion of the right retro-orbital region (**A:** contrast-enhanced T1-weighted axial magnetic resonance imaging). Percutaneous biopsy was performed through a right periorbital approach (**B,C**). Histopathological examination revealed a melanocytoma.

*Periorbital*-For target lesions in the retro-orbital or periorbital space (Figure 4).

#### Infrahyoid

*Anterolateral*-For target lesions in the retrotracheal and paraesophageal regions, as well as the anterior paravertebral spaces, lower cervical paravertebral spaces, and intervertebral discs.

*Posterolateral*-For target lesions in the paraspinal portions of the paravertebral spaces, as well as the retropharyngeal and posterior cervical spaces.

*Posterior*-For target lesions in the spinous processes, laminae, and articular processes of the lower cervical vertebrae, as well as the lateral paraspinal portions of the paravertebral space.

### Statistical analysis

Summary data were reported as means and standard deviations, as median, minimum, and maximum values, or as absolute and relative frequencies, and graphs were plotted. The variables of interest were tabulated and presented as absolute and relative frequencies. Unpaired Student’s *t*-tests were used in order to test for statistical significance of the differences in means between quantitative variables. To compare categorical data, we used the chi-square test or Fisher’s exact test, as appropriate. The Shapiro-Wilk test was used in order to determine the normality of distribution. All statistical analyses were performed with the IBM SPSS Statistics software package, version 20.0 (IBM Corp., Armonk, NY, USA). The significance level was set at 5% for all analyses.

For the analysis of diagnostic accuracy, sensitivity, specificity, positive predictive value (PPV), and negative predictive value (PPV), the biopsy results were compared with the final diagnosis of each case. The final diagnoses were determined by a combination of histological examination and clinical follow-up, including new biopsies, surgical resection, and other treatments. In patients who underwent surgical resection, we compared the results of the histological examination of the material obtained by percutaneous biopsy with those of the histological examination of the surgical specimens (the gold-standard method). Proportions were provided, along with binomial exact 95% confidence intervals (CIs), the “exact” Clopper-Pearson CIs being used for sensitivity and specificity, whereas the standard logit CIs were used for PPV and NPV.

## RESULTS

### Patient characteristics

We evaluated 74 biopsies performed in 68 patients (four patients underwent two biopsies, and one underwent three biopsies). Of the 68 patients evaluated, 40 (58.8%) were male. The mean age was 55.6 ± 15.1 years (range, 14-83 years).

The range of primary diagnoses was broad, led by squamous cell carcinoma of the head and neck (SCCHN; n = 26), followed by unknown primary tumor (n = 21), thyroid cancer (n = 7), breast cancer (n = 5), gynecological cancer (n = 4), multiple myeloma (n = 3), and other malignancies (n = 8). Most of the patients (67.6%) had previously undergone some form of treatment (surgery, chemotherapy, radiotherapy, or a combination of those).

### Biopsy characteristics

*Target lesions*

Of the 74 biopsies performed, 59 (79.7%) targeted masses in the suprahyoid space and 15 (20.3%) targeted masses in the infrahyoid space. The mean maximum diameter of the masses was 35.6 ± 11.0 mm (range, 11-128 mm). Intravenous injection of iodinated contrast media prior to CT acquisition was necessary for better delineation of target lesions in only five biopsies (6.8%).

*Technique*

The coaxial system with an 18-gauge core needle was used in 78.4% of the biopsies, whereas 16-gauge and 20-gauge needles were used in 10% and 2%, respectively. When there was bone involvement, larger needles were used, 8-gauge and 11-gauge needles being used in 8% and 2% of the biopsies, respectively.

A paramaxillary approach was used in 32.4% of the biopsies, whereas retromandibular and periorbital approaches were used in 21.6% and 14.9%, respectively. Less commonly, the anterolateral, infrazygomatic, anterior, and posterior approaches were used, and direct access was used for large masses in which the target exceeded known anatomical boundaries.

*Complications*

Five patients (6.8%) developed complications after biopsy. Three of those patients had small local hematomas, with no immediate clinical repercussion. All of those hematomas appeared stable or were reduced in size on a follow-up CT performed at 2 h after biopsy. One patient developed facial nerve palsy, which resolved completely within 3 h after the biopsy. Another patient complained of persistent pain, which was reported at a follow-up visit to the physician who had ordered the procedure.

The presence of complications did not show a statistically significant association with any clinical factor (age, gender, diagnosis, or previous treatment), radiological factor (mass size or location), or procedure-related factor (needle gauge or approach).

### Histopathological findings

Sufficient material for histological analysis was obtained in all cases. In 38 (51.4%) of the biopsies evaluated, the histological analysis revealed malignancy. All of the affected patients were treated accordingly and received specific therapy, including chemotherapy, surgery, or radiotherapy, as appropriate.

Of the 36 lesions diagnosed as benign in the histological analysis, 20 (55.6%) did not undergo further biopsy or surgery, and the affected patients remained free of signs or symptoms of disease related to the same lesion during the clinical and imaging follow-up period; 11 (30.6%) were surgically resected, the analysis of the surgical specimen resulting in confirmation of the benign status in ten and a diagnosis of malignancy in one; three (8.3%) underwent a repeat biopsy, which confirmed the initial diagnosis (no signs or symptoms of disease being observed at the biopsy site) in two; one (2.8%) had discordant results and received a diagnosis of desmoplastic fibroblastoma on a repeat biopsy, that diagnosis being confirmed after analysis of the surgical specimen; one (2.8%) was found to have enlarged on follow-up imaging at 8 months after the initial biopsy, and a new percutaneous biopsy confirmed the diagnosis of malignancy; and one (2.8%) was diagnosed as jugular paraganglioma and referred for radiotherapy after refusal of the surgical treatment proposed. Therefore, three (4.1%) of the 74 initial biopsies were considered to have yielded false-negative results.

Overall, CT-guided percutaneous biopsy of head and neck masses showed a sensitivity of 92.7%, a specificity of 100%, and a diagnostic accuracy of 96.0% (Table 1).

**Table 1:** Comparison between the results of CT-guided percutaneous coreneedle biopsies of head and neck masses (n = 74) and the final (histopathological) diagnosis.

Biopsy result	Final diagnosis		Total
	Malignant	Benign	
Malignant	38	0	38
Benign	3	33	36
Total	41	33	74

Sensitivity: 92.7% (95% CI: 80.1-98.5%); Specificity: 100% (95% CI: 89.4­100%); PPV: 100%; NPV: 91.7% (95% CI: 78.7-97.0%); Accuracy: 96.0% (95% CI: 88.6-99.2%).

### Suprahyoid masses

Lesions located in the suprahyoid region were investigated in 59 biopsies performed in 54 patients, of whom 32 (59.3%) were male. In this subgroup, the mean patient age was 53.6 ± 15.5 years (range, 14-83 years), and the most common primary diagnosis was SCCHN. Of the 54 patients in the suprahyoid group, 36 (66.7%) had previously undergone treatment. The paramaxillary, retromandibular, and periorbital approaches were used in 24 (40.7%), 16 (27.1%), and 11 (18.6%) of the biopsies, respectively, other approaches being used in the remaining eight biopsies (13.6%).

Complications occurred after four (6.8%) of the biopsies performed in the suprahyoid group. As in the sample as a whole, the occurrence of complications in the suprahyoid group showed no statistically significant associations with clinical, radiological, or procedure-related factors. In 30 (50.8%) of the 59 suprahyoid biopsies, the histological findings were consistent with malignancy. Two (6.8%) of those 30 cases were considered false-negatives. Among the 59 biopsies in the suprahyoid group, CT-guided percutaneous biopsy had a sensitivity of 93.3%, a specificity of 100%, and a diagnostic accuracy of 96.6% (Table 2).

**Table 2:** Comparison between the results of CT-guided percutaneous coreneedle biopsies of suprahyoid head and neck masses (n = 59) and the final (histopathological) diagnosis.

Biopsy result	Final diagnosis		Total
	Malignant	Benign	
Malignant	28	0	28
Benign	2	29	31
Total	30	29	59

Sensitivity: 93.3% (95% CI: 77.9-99.2%); Specificity: 100% (95% CI: 88.0­100%); PPV: 100%; NPV: 93.6% (95% CI: 79.2-99.6%); Accuracy: 96.6% (95% CI: 88.3-99.6%).

### Infrahyoid masses

Lesions located in the infrahyoid region were investigated in 15 biopsies performed in 14 patients, of whom seven (50.0%) were male. In this subgroup, the mean patient age was 63.5 ± 11.2 years (range, 46-82 years), and, as in the suprahyoid group, the most common primary diagnosis was SCCHN. Of the 14 patients in the suprahyoid group, 11 (78.6%) had previously undergone treatment. The most common was anterolateral approach was used in eight (53.3%) of the biopsies, followed by the anterior approach, in three (20.0%), the posterior approach, in three (20.0%), and direct access, in one (6.7%). Complications occurred after only one (6.7%) of the biopsies performed in the infrahyoid group. As in the sample as a whole, the occurrence of complications in the infrahyoid group showed no statistically significant associations with clinical, radiological, or procedure-related factors. In eight (53.3%) of the 15 infrahyoid biopsies, the histological findings were consistent with malignancy. One (14.3%) of those eight cases was considered a false-negative. Among the 15 biopsies in the infrahyoid group, CT-guided percutaneous biopsy had a sensitivity of 87.5%, a specificity of 100%, and a diagnostic accuracy of 93.3% (Table 3).

**Table 3:** Comparison between the results of CT-guided percutaneous coreneedle biopsies of infrahyoid head and neck masses (n = 15) and the final (histopathological) diagnosis.

Biopsy result	Final diagnosis		Total
	Malignant	Benign	
Malignant	7	0	7
Benign	1	7	8
Total	8	7	15

Sensitivity: 87.5% (95% CI: 47.4-99.7%); Specificity: 100% (95% CI: 59.0­100%); PPV: 100%; NPV: 87.5% (95% CI: 52.8-97.8%); Accuracy: 93.3% (95% CI: 68.0-99.8%)

## DISCUSSION

In the present study, we have reported the results of 74 CT-guided percutaneous core-needle biopsies performed in patients with suspicious masses in the deep spaces of the head and neck, a sample larger than those evaluated in previous studies^(1,10-12)^. Sufficient material for histological analysis was obtained in all cases. Our analysis of accuracy took into account biopsy results, the findings of the histopathological examination of surgical specimens (in patients who underwent surgery), and clinical follow-up data collected during the study period.

In three patients, the initial biopsies produced false-negative results, and two of those patients received a definitive diagnosis after a second biopsy. One of those two, a 29-year-old male, underwent biopsy of a mass in the left retropharyngeal space, which was initially diagnosed as striated muscle tissue and hyalinized fibrous tissue. Because of the absence of a known history of cancer, the decision was made to repeat the biopsy after a brief interval. In the second biopsy, the histological findings were suggestive of desmoplastic fibroblastoma. Surgical resection was recommended, and the histological analysis confirmed the diagnosis of desmoplastic fibroblastoma, a rare, benign tumor, which-like any soft-tissue tumor-certainly represents a challenge for the pathologist due to the histological similarity between fibroblastic and myofibroblastic proliferation^(13)^. The other patient who received a definitive diagnosis after the second biopsy had a history of squamous cell carcinoma of the larynx, which had been treated with chemotherapy and radiotherapy, was referred for biopsy of the right anterior commissure of the larynx after positron-emission tomography/CT (PET/CT) showed slight enlargement and low fluorodeoxyglucose uptake. In that patient, the first biopsy was negative, and the diagnosis was one of fibrosis and granulomatous reaction. Nonetheless, serial follow-up imaging was performed. At 8 months after the initial biopsy, the imaging showed signs of local disease progression, with further enlargement of the lesion, which was actually detectable on plain CT at the time of the second biopsy, at which point a diagnosis of moderately differentiated SCCHN was established. In that case, the negative result of the first biopsy may be attributable to the small size of the lesion at the time. The third false-negative result was discovered after surgical resection of the mandible, which yielded a final diagnosis of osteosarcoma. In that patient, the initial biopsy, of a soft-tissue mass near the left mandible, suggestive of neoplastic infiltration on CT, had yielded a report of fibrous/connective tissue with foreign-body granulomas and focal areas of hemorrhage, possibly related to a previous surgical procedure, which did not include the tumor itself.

Image-guided biopsies have been the subject of a number of recent articles in the radiology literature of Brazil^(14-16)^. The accuracy of biopsy for a final diagnosis in our sample was calculated as 96%. This rate is similar to those reported in other studies, such as that conducted by Wu et al.^(1)^, who achieved 96.4% accuracy for core-needle biopsies of masses in the suprahyoid space in patients with a known history of head and neck neoplasms. That is comparable to the 96.6% observed in our subgroup of 59 biopsies of masses in the suprahyoid space. In another study, Wu et al.^(11)^ found that core-needle biopsy resulted in a correct diagnosis in 9 of 10 patients with masses at the base of the skull and no known history of cancer. In a study of 15 biopsies of the deep spaces of the face and skull base in a mixed patient population (i.e., with and without a known history of cancer, as in the present study), Connor et al.^(10)^ reported a diagnostic accuracy of 87%. In a more recent study, Gao et al.^(17)^ reported a 90.3% accuracy for PET/CT-guided core-needle biopsy of deep head and neck lesions.

Factors that may be associated with lower success rates in obtaining material were described in the study conducted by Cunningham et al.^(18)^, who reported correct diagnoses in 73% of 22 CT-guided core-needle biopsies of the deep face and skull base. In that study, the institutional biopsy protocol involved the use of a semi-automated biopsy device with an 18-gauge needle, which yielded an average of two fragments per procedure, and the coaxial technique, as described by Connor et al.^(10)^ and Wu et al.^(1,11)^, was employed in only five of the 22 cases evaluated. The authors found that, in 50% of cases in which specimens were insufficient for diagnosis, only one fragment had been obtained, compared with two to three fragments in the cases in which the specimens were sufficient for diagnosis. Those authors also reported that a correct diagnosis was associated with the mean axial diameter of the mass, which was 4.5 cm in the group with a diagnosis and 2.0 cm in the group without. No such associations were observed in the present study, because all of the specimens were considered sufficient for diagnosis.

Complications after head and neck biopsies are rare and are usually of little clinical significance^(1,8,11,19)^. They most commonly consist of pain, vasovagal reaction, infections, and minor bleeding. According to some authors, a history of surgery or radiotherapy increases the odds of vascular complications^(11)^. Cranial nerve injury leading to motor or sensory deficits is uncommon in the literature^(10)^. In biopsies of orbital masses, retrobulbar hemorrhage is the most common complication, although it is typically minor and reabsorbed with no ill effects^(20)^. Severe complications, which include injury to the globe and optic nerve, are rare in image-guided procedures, especially when performed by experienced radiologists^(17)^. Familiarity with the noble structures of the region, especially when dealing with masses in the deep spaces of the head and neck, together with adequate planning, are essential to make the procedure feasible; when done properly, it is an excellent alternative to conventional surgical techniques^(8)^.

Probably due to the small number of complications in relation to the large number of procedures performed, both in the overall sample as a whole, as well as in the suprahyoid and infrahyoid subgroups, we found that the development of complications was not associated with needle gauge, mass location/size, history of cancer, previous treatment, or histological diagnosis. All three cases of local bleeding were self-limited, with no increase in size on a follow-up CT performed before hospital discharge (usually 2 h after the procedure). One case of transient facial palsy occurred immediately upon infiltration of 1% lidocaine, without a vasoconstrictor, for local anesthesia. The patient had undergone biopsy of a mass in the right parapharyngeal space through the ipsilateral paramaxillary approach, and the final diagnosis was lipoma. The patient remained under observation for 2 h and was discharged with no neurologic deficits. Wu et al.^(1)^ reported a similar incidence of complications (7.1%) and detected no statistical association between the occurrence of complications and needle gauge. In that study, the authors reported one case of local hematoma and one case of transient facial nerve palsy, both of which were considered minor complications, because they did not require hospital admission^(1)^. Three other such studies^(10,11,18)^, perhaps due to their smaller sample sizes (10, 18, and 22 patients, respectively), did not report any complications after biopsy.

One noteworthy finding of the present study is that the use of intravenous contrast to improve the delineation of the target lesion in a pre-biopsy CT was not associated with diagnostic accuracy or the complication rate. However, contrast was used much less frequently in our study than in previous studies, in which it was used in up to 75% of cases^(1)^.

Our study has some limitations, including those inherent to a retrospective design. In addition, the gold-standard diagnostic method-surgical resection and subsequent histological examination of the target lesion-was employed in relatively few cases (34.2% in the malignant outcome group and 30.6% in the benign outcome group). This may have occurred because the majority of patients (69.9%) had a known history of cancer, which facilitates acceptance (by the requesting physician) of a diagnosis of recurrence or metastasis through percutaneous biopsy alone. Another important factor that should be taken into account is that many patients were no longer considered candidates for surgery even prior to the biopsy result, either for technical reasons (such as multiple prior surgeries and local irradiation) or clinical reasons (such as advanced age, advanced disease stage, and prohibitive comorbidities). In this context, the method commonly used by other authors^(1,10,11)^ to confirm that the biopsied lesions are genuinely benign is clinical and imaging follow-up evaluations, which should demonstrate absence of disease progression at the biopsy site. The duration of follow-up has been quite variable in the literature, depending on the study design; follow-up periods are sometimes quite short, especially for patients enrolled near the end of data collection, which makes this method unreliable^(11)^. 

Because our sample was heterogeneous, including biopsies of masses in the suprahyoid and infrahyoid spaces (including retro-orbital masses), of lymph nodes at various cervical levels, and of suspicious masses at a wide variety of sites, including cartilage, bone, soft tissues, the base of the tongue, the tonsils, and even the esophagus-in patients with and without a known history of cancer, treated or not for underlying disease-it is not feasible to compare our results with those in the literature, because previous studies have tended to focus on lesions with similar characteristics. For instance, Wu et al.^(1)^ included only lesions in the suprahyoid space in patients previously treated for head and neck cancer; Connor et al.^(10)^, Wu et al.^(11)^, and Cunningham et al.^(18)^ included only patients who had never undergone any form of previous treatment. However, we can and should regard our findings as a reference that could serve as a source for further, more specific analyses (such as our own subgroup analysis of suprahyoid and infrahyoid biopsies), thus facilitating future research and comparisons across studies.

In conclusion, the high accuracy and low complication rate of CT-guided percutaneous core-needle biopsy of suspicious head and neck masses suggest that, in the hands of experienced interventional radiologists, it is a safe, effective method for obtaining tissue samples for definitive histological diagnosis.
